# Responses of Polyamine-Metabolic Genes to Polyamines and Plant Stress Hormones in Arabidopsis Seedlings

**DOI:** 10.3390/cells10123283

**Published:** 2021-11-24

**Authors:** Yusaku Yariuchi, Takashi Okamoto, Yoshiteru Noutoshi, Taku Takahashi

**Affiliations:** 1 Graduate School of Natural Science and Technology, Okayama University, Okayama 700-8530, Japan; yarioka@s.okayama-u.ac.jp (Y.Y.); takashi_okamoto@cc.okayama-u.ac.jp (T.O.); 2 Graduate School of Environmental and Life Science, Okayama University, Okayama 700-8530, Japan; noutoshi@cc.okayama-u.ac.jp

**Keywords:** abscisic acid, Arabidopsis, jasmonate, polyamine metabolism, salicylic acid

## Abstract

In plants, many of the enzymes in polyamine metabolism are encoded by multiple genes, whose expressions are differentially regulated under different physiological conditions. For comprehensive understanding of their regulation during the seedling growth stage, we examined the expression of polyamine metabolic genes in response to polyamines and stress-related plant hormones in *Arabidopsis thaliana*. While confirming previous findings such as induction of many of the genes by abscisic acid, induction of arginase genes and a copper amine oxidase gene, *CuAOα3*, by methyl jasmonate, that of an arginine decarboxylase gene, *ADC2*, and a spermine synthase gene, *SPMS*, by salicylic acid, and negative feedback regulation of thermospermine biosynthetic genes by thermospermine, our results showed that expressions of most of the genes are not responsive to exogenous polyamines. We thus examined expression of *OsPAO6*, which encodes an apoplastic polyamine oxidase and is strongly induced by polyamines in rice, by using the promoter-GUS fusion in transgenic Arabidopsis seedlings. The GUS activity was increased by treatment with methyl jasmonate but neither by polyamines nor by other plant hormones, suggesting a difference in the response to polyamines between Arabidopsis and rice. Our results provide a framework to study regulatory modules directing expression of each polyamine metabolic gene.

## 1. Introduction

Polyamines function in various aspects of plant development and stress responses. In land plants, many of the enzymes in polyamine biosynthetic and catabolic pathways are encoded by multiple genes, which show differential expression patterns under different physiological conditions and in different tissues. In *Arabidopsis thaliana*, there are two genes encoding arginine decarboxylase (ADC), a key enzyme for putrescine biosynthesis, *ADC1* and *ADC2*. *ADC1* shows a constitutive expression and is also responsive to cold and bacterial pathogen infection, while *ADC2* is upregulated by abiotic stresses such as salt, drought, cold, and wounding [1,2,3,4,5,6,7]. Agmatine produced by ADC is converted to putrescine by two sequential enzymes of agmatine iminohydrolase (AIH) [8] and N-carbamoylputrescine amidohydrolase (CPA) [9] or in a single step mediated by agmatinase. The Arabidopsis genome has two genes encoding a dual functional arginase/agmatinase, *ARGAH1* and *ARGAH2*. Expression of both genes is co-regulated with *ADC2* in response to abscisic acid (ABA), methyl jasmonate (MeJA), drought, cold, and high salinity, suggesting that the agmatinase pathway may be a major contributor to putrescine biosynthesis under stress conditions [10]. ABA also upregulates the expression of a gene for spermine synthase, *SPMS* [11]. On the other hand, *ACL5* encoding thermospermine synthase is specifically expressed in xylem precursor cells and plays a key role in repressing xylem differentiation. *ACL5* expression is upregulated by auxin and downregulated by thermospermine [12]. Biosynthesis of spermidine, spermine, and thermospermine requires decarboxylated S-adenosyl methionine as an aminopropyl donor, which is supplied by S-adenosyl methionine decarboxylase (SAMDC/AdoMetDC). Among four genes encoding SAMDC/AdoMetDC in Arabidopsis, *SAMDC1*, *SAMDC2*, and *SAMDC3*, contain conserved upstream open-reading frames (uORFs) within the 5′ leader region of each mRNA. The uORFs of *SAMDC1* have been shown to play a regulatory role in the translation of the main ORF in response to cellular polyamine levels [13]. A recent study has reported that expression of *ADC1*, *SAMDC2*, *SAMDC4*, and two genes for spermidine synthase, *SPDS1* and *SPDS2*, are drastically induced during cytokinin-induced direct shoot regeneration from root primordia, suggesting the importance of spermidine in plant regeneration [14].

Plant polyamine catabolism is mediated by copper amine oxidase (CuAO), which mainly oxidizes putrescine, and polyamine oxidase (PAO). These amine oxidases participate in important physiological processes through the production of hydrogen peroxide [15,16,17,18]. Arabidopsis contains five genes for PAO and eight genes for CuAO. *CuAOγ1* and *CuAOζ*, which encode an extracellular and peroxisomal CuAO, respectively, are upregulated by ABA and salicylic acid (SA), while *CuAOα3* encoding a peroxisomal enzyme shows a marked induction by methyl jasmonate (MeJA) and wounding [19]. Another study revealed a transient increase for several hours in the expression of *CuAOα2*, *CuAOα3*, *CuAOγ1*, and *CuAOγ2* by auxin [20] and that of *CuAOδ* by ABA [21]. *CuAOα3*, *CuAOγ1*, and *CuAOγ2* were also shown to be upregulated by putrescine [20]. On the other hand, *PAO1*, *PAO3*, and *PAO4* expressions are increased by treatment with salicylic acid for 24 h [22,23]. *PAO3* expression is also responsive to ABA and MeJA [22]. A transgenic study revealed that overproduction of thermospermine by ectopic expression of *ACL5* results in an increased expression of *PAO1*, *PAO3*, and *CuAOβ* [24]. Most land plants, except the superasterid clade and a part of the superrosid clade, including Arabidopsis in eudicots, contain extracellular PAOs [25], and they may play a protective role against pathogens or wounding by generating hydrogen peroxide and amino aldehydes [15,16,17,18]. In maize, extracellular PAO activity has been shown to be involved in peroxidase-mediated wall stiffening events during wound healing [16]. Expressions of the genes encoding extracellular PAOs in rice were shown to be induced by polyamines [26] and MeJA [27].

Taken together, these studies indicate that different genes for the same metabolic functions play a role in different physiological or environmental conditions. A genome-wide transcriptome analysis of polyamine metabolic genes in tomato leaves reveals a more pronounced role of polyamines in cold stress acclimation than in heat stress tolerance [28]. Here, towards comprehensively understanding the mechanism of differential regulation of polyamine metabolic genes in Arabidopsis, we focused on the response of these genes to stress-related plant hormones and polyamines during the seedling stage.

## 2. Materials and Methods

### 2.1. Chemicals

All polyamines used in this study were hydrochloride salts. Putrescine, spermidine, and spermine were purchased from Nakalai Tesque (Kyoto, Japan). Thermospermine was purchased from Santa Cruz Biotechnology (Santa Cruz, CA, USA).

### 2.2. Plant Growth Conditions

The Columbia (Col-0) accession of *Arabidopsis thaliana* was used as wild type. Seeds were surface-sterilized with bleach solution containing 0.01% (*w/v*) Triton X-100 for 3 min and rinsed three times in sterile water. Approximately 30 seeds were sown in 25 mL of half-strength MS (Wako, Tokyo, Japan) liquid media at pH 5.7 containing 1% sucrose and cultured for 7 days on rotary shakers at 22 °C under 16 h light/8 h dark conditions. For treatment with polyamines or plant hormones, seedlings were further incubated in the presence of each compound for 6 h unless otherwise stated. Polyamine concentrations were chosen based on our preliminary results on minimum concentration levels toxic to the seedling growth and previous studies [29].

### 2.3. RNA Extraction and Expression Analyses

Total RNA was extracted from whole seedlings by the SDS-phenol method followed by LiCl precipitation [30] and reverse-transcribed using a PrimeScript RT reagent kit (Takara, Kyoto, Japan) with the oligo dT primer. PCR reactions were performed using a KAPA SYBR FAST qPCR kit (KAPA Biosystems, Woburn, MA, USA) and the Thermal Cycler Dice TP760 (Takara) with gene-specific primers (Supplementary Table S1). *ACTIN8* (At1g49240) was used to normalize the reaction. *UBQ10* (At4g05320) was used as another reference gene and confirmed to show no significant difference from *ACT8* in the response to the chemicals examined (Table 1 and Table 2). For each plant treatment, RNA was prepared three times, and each sample was duplicated in PCR reactions.

### 2.4. T-DNA Construction and Plant Transformation

For the *OsPAO6* promoter-driven expression of the GUS reporter gene, a 990-bp genomic fragment upstream from the 1st exon of *OsPAO6* was amplified by PCR with primers OsPAO6pro-FCl (5′-ATCGA TGAAG CTGCC ATCGC CAG-3′) and OsPAO6pro-RBm (5′-GGATC CCTTC TTGGC ACGAG AATG-3′), cloned into a pGEM-T Easy vector (Promega, WI, USA), and then transferred to *Cla*I-*Bam*HI sites of Ti-plasmid vector pBI101 (Clontech, CA, USA). The resulting Ti plasmid construct was introduced into *Agrobacterium tumefaciens* C58C1 by electroporation. Arabidopsis plants were transformed using the floral dip method [31]. Transgenic lines were selected on kanamycin and confirmed by PCR using the T-DNA-specific primers, pBI-ClaF (5′-GTCGC TACTG ATTAC GG-3′) and GUS-R (5′-TCACG GGTTG GGGTT TCTAC-3′). Three independent homozygous lines carrying single copy of the transgene were further selected based on the segregation ratio of kanamycin-resistant plants in subsequent generations.

### 2.5. GUS Assays

Fluorometric assay of the bacterial β-glucuronidase (GUS) activity was performed as described previously [32]. The fluorescence was measured with an RF-5300PC spectrofluorophotometer (Shimadzu, Kyoto, Japan). Total protein content was measured by using the Bradford assay (BioRad, Hercules, CA, USA).

### 2.6. Statistics

All statistical tests were performed using Student’s *t*-test (* *p* < 0.05, ** *p* < 0.01).

## 3. Results

### 3.1. Responses of Polyamine Metabolic Genes to Polyamines

In this study, we examined expressions of all 27 genes directly involved in polyamine biosynthesis and degradation in 7-day-old seedlings of Arabidopsis grown in liquid media. Although we could not detect expression of *CuAOα1* at the seedling stage, most of the genes showed no significant change in transcript levels after treatment for 6 h with 5 mM putrescine, 1 mM spermidine, and 0.5 mM spermine or 0.1mM thermospermine (Table 1). The exceptions were *ADC2*, *ACL5*, *SAMDC4/BUD2*, and *PAO2*. In agreement with previous studies [12,33], expressions of *ACL5* and *SAMDC4/BUSHY AND DWARF2* (*BUD2*), both of which are specifically expressed in the vasculature and involved in thermospermine biosynthesis, were reduced by thermospermine. *PAO5* encoding thermospermine oxidase was not responsive to thermospermine. *ADC2* expression was reduced by spermidine but rather promoted by putrescine. Expression of *PAO2*, which is mainly involved in spermidine oxidation, was upregulated by spermidine, but *PAO3*, which also functions in spermidine oxidation, showed no significant response to spermidine.

### 3.2. Responses of Polyamine Metabolic Genes to Plant Stress Hormones

We next examined the response of these genes to stress-related plant hormones. Seedlings were incubated for 6 h with each hormone (Table 2). Treatment with an auxin, indole acetic acid (IAA), activated expression of not only *ACL5* and *SAMDC4/BUD2* but also *ADC2*, *PAO1*, *PAO3*, and *PAO5*. *CuAOα2* and *CuAOα3* were downregulated by IAA. No genes showed significant changes in response to kinetin under our experimental conditions. Treatment with ABA activated expression of 16 genes involved in the biosynthesis or catabolism of polyamines. The genes that showed no significant response to ABA were *ADC1*, *SPDS1*, *ACL5*, *SAMDC3*, *SAMDC4/BUD2*, *PAO5*, *CuAOα2*, *CuAOα3*, *CuAOβ*, *CuAOγ2*, and *CuAOζ*. Treatment with amino cyclopropane carboxylate (ACC) as a precursor of ethylene also upregulated expression of *ADC2* but downregulated expression of *CuAOα2*. Treatment with MeJA caused a drastic increase in the expression of *ARGAH2* and a several-fold increase in the expression of *ARGAH1* and *CuAOα3*, as reported previously [19,34], and also of *PAO3*. The results also revealed that sodium salicylate (NaSA), which was used as the source of salicylic acid, upregulated expression of *ADC2*, *AIH*, *SPMS*, *SAMDC1*, and *PAO1* but downregulated expression of *SAMDC2*, *CuAOα2*, *CuAOα3*, and *CuAOγ2.*

**Figure 1:**
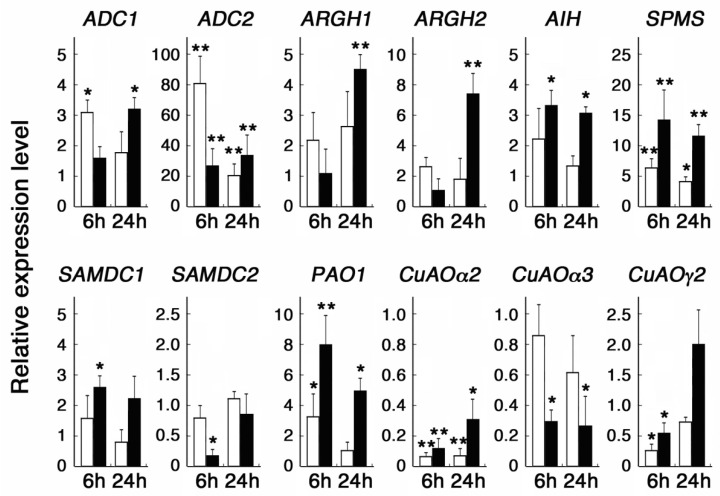
Effect of MeSA and NaSA on polyamine metabolic genes. Seven-day-old seedlings grown in liquid MS solution were further incubated with 1mM MeSA (white bars) or NaSA (black bars) for 6 and 24 h. Transcript levels relative to the control of no SA treatment are shown. Data are expressed as mean values ± SD (*n* = 6). Asterisks indicate the statistical significance analyzed with Student’s *t*-test, (* *p* < 0.05, ** *p* < 0.01).

Salicylic acid has been shown to be converted at the primary site of pathogen infection to methyl salicylate (MeSA), which in turn acts as a mobile signal and is again reversed at distant tissues to induce systemic acquired resistance [35]. We thus compared the responses of the above-described NaSA-responsive genes to sodium and methyl SA. Among the genes examined, *ADC2* showed the strongest induction after 6 h treatment with MeSA, while it also showed a more than twenty-fold increase in the transcript level after 6 and 24 h treatment with NaSA (Figure 1). *AIH*, *SPMS*, and *PAO1* also showed increased transcript levels after 6 h treatment with MeSA but to a lesser degree than the treatment with NaSA (Figure 1). *SAMDC1* responded slightly to NaSA but showed no significant response to MeSA (Figure 1). We also found that *ARGH1* and *ARGH2* transcript levels were upregulated after 24 h treatment with NaSA, although these were not responsive to the 6 h treatment and were only slightly upregulated by treatment with MeSA (Figure 1). On the other hand, *SAMDC2* and *CuAOα3,* whose transcript levels were reduced by NaSA, showed no significant response to MeSA, while *CuAOα2* and *CuAOγ2* showed reduced transcript levels after 6 h treatment with both MeSA and NaSA (Figure 1).

### 3.3. OsPAO6 Promoter Is Responsive to MeJA in Arabidopsis

**Figure 2:**
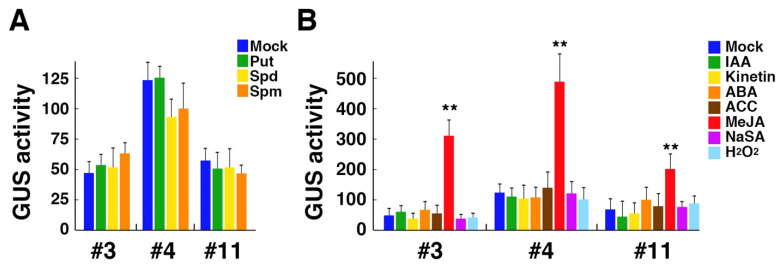
Effect of polyamines and stress-related plant hormones on the GUS activity in three transgenic Arabidopsis lines, #3, #4, and #11, carrying the *OsPAO6* promoter-GUS construct. (**A**) The relative GUS activity of 7-day-old seedlings after treatment with 5 mM putrescine (Put), 1 mM spermidine (Spd), or 0.5 mM spermine (Spm) for 24 h. (**B**) The relative GUS activity of 7-day-old seedlings after treatment with 10 μM IAA, 5 μM kinetin, 1 μM ABA, 10 μM ACC, 2 μM MeJA, 1 mM NaSA, or 0.3% hydrogen peroxide for 24 h. Data are expressed as mean values ± SD (n = 3). Asterisks indicate the statistical significance analyzed with Student’s *t*-test (** *p* < 0.01).

Although all genes related to polyamine metabolism showed no significant change in the transcript level after spermine treatment in our experiments, we have recently found that expressions of *OsPAO2* and *OsPAO6*, both of which encode extracellular enzymes, are drastically induced by spermine in rice [26]. We thus generated transgenic Arabidopsis lines with the GUS reporter gene under the control of a 1.1-kb promoter of *OsPAO6* and examined whether the GUS expression is upregulated by spermine treatment or not. From more than 10 independent transgenic lines obtained, we selected three transgenic lines that showed a 3:1 ratio for kanamycin resistance in T2 generation and were established as homozygous T-DNA lines in the progeny. These lines showed no significant increase in the GUS activity after one-day treatment with putrescine, spermidine, and spermine (Figure 2A). We further examined the GUS activity after treatment with the above-examined plant hormones and found that MeJA had a strong inducing effect on the GUS activity while other hormones had no effect in these lines (Figure 2B). We also examined the effect of hydrogen peroxide, which is a product of polyamine oxidation, on the GUS activity but found no significant effect (Figure 2B).

## 4. Discussion

While much attention has been paid to the role of each polyamine in diverse plant physiological processes, regulatory mechanisms of transcriptional or post-transcriptional response of polyamine metabolic genes to polyamines or plant hormones have not been fully addressed. This prompted us to investigate the expression of these genes comprehensively in Arabidopsis seedlings. In this study, we focused on a fixed experimental condition in terms of duration and concentration of chemical treatments. Thus, the possibility should not be excluded that expression of some genes could show a fast and transient response to a certain polyamine or plant hormone, or also be responsive to even much lower concentration of polyamines. Our results revealed that the transcript levels of most of the genes involved in the synthesis and oxidative degradation of polyamines are not altered by 6 h treatment with putrescine, spermidine, spermine, and thermospermine. The exceptions are *ADC2*, *PAO2*, *ACL5*, and *SAMDC4/BUD2*, the latter two of which have been shown to be under negative feedback regulation by thermospermine [12,33]. Transcription of *ACL5* is interfered with basic helix-loop-helix proteins of the SAC51 family whose translation is enhanced by thermospermine [36,37]. It remains to be examined whether *SAMDC4/BUD2* is under the same regulation or not.

The *ADC2* transcript level was increased by putrescine but reduced by spermidine. Because the transcript levels of *ARGAH1*, *ARGAH2*, and *CPA*, the genes for the last step of putrescine biosynthesis, were not altered by putrescine, only *ADC2* may be regulated by positive feedback from putrescine or by the balance between putrescine and spermidine levels. Putrescine treatment has recently been shown to lead to local SA biosynthesis in Arabidopsis [38], while *ADC2* expression was strongly induced by both NaSA and MeSA (Figure 1). It is thus possible that the *ADC2* induction is caused by putrescine-dependent SA accumulation. A previous study using the GUS reporter gene has shown that the difference in the response to stresses between *ADC1* and *ADC2* is attributable to that of the promoter activity [6]. In an earlier work, a short uORF present in the 5′ leader of an *ADC* mRNA in carnation has been suggested to act in the translational regulation of the main coding sequence [39]. A recent study revealed the presence of the conserved non-AUG-initiated uORF in the Arabidopsis *ADC2* mRNA [40]. Future investigations are expected to clarify whether the response of *ADC2* to polyamines and other stimuli involves uORF-mediated translational regulation or not.

Most of the genes involved in polyamine oxidation were not responsive to exogenous polyamines in Arabidopsis seedlings. This suggests no tight correlation between cellular polyamine concentrations and regulation of polyamine-catabolic genes. Considering the toxic effect of the catalytic products of polyamine oxidation such as hydrogen peroxide and amino aldehydes, it is reasonable that expressions of these genes are not altered drastically but finely tuned depending upon physiological conditions. However, a previous study has revealed that expressions of *PAO1*, *PAO3*, and *PAO5* are slightly increased by thermospermine and spermine [24]. Another study has detected an increase in expressions of *CuAOα3*, *CuAOγ1*, and *CuAOγ2* by putrescine, although their time courses of induction and recovery are not the same [20]. In tomato, which also lacks extracellular PAOs, expressions of *SlPAO2*, *SlPAO3*, and *SlPAO4*, which may be orthologous to *PAO2*, *PAO3*, and *PAO4* of Arabidopsis, respectively, are induced by polyamines [41]. The difference of the responses observed between studies might be due to different experimental conditions including the duration of chemical treatment and growth media. *PAO2*, *PAO3*, and *PAO4* contain conserved uORFs, suggesting again the involvement of translational regulation in polyamine metabolism. The GUS reporter activity under the control of the CaMV 35S promoter fused with the 5′ leader region of *PAO2* has been shown to be induced by polyamines in transgenic Arabidopsis seedlings [42,43].

Expressions of *OsPAO2* and *OsPAO6*, encoding extracellular PAOs in rice, are strongly induced by polyamines [26,27]. The 5′ leaders of these genes have no conserved uORFs [44]. The *OsPAO6* promoter-GUS reporter gene showed no response to polyamines in transgenic Arabidopsis seedlings, while it was responsive to MeJA, indicating that the response of the *OsPAO6* promoter to polyamines is uncoupled from that to MeJA. This suggests a difference in transcriptional regulation of *PAO* genes between rice and Arabidopsis, although it remains to be determined whether this reporter construct responds to polyamines in rice or not. No response in Arabidopsis might also reflect the absence of apoplastic PAOs in Arabidopsis. It is possible that the response to exogenously supplied polyamines in rice involves their catalytic products generated by apoplastic PAOs. More detailed and comparative studies of the *OsPAO6* promoter in rice and Arabidopsis will be needed to elucidate the precise molecular mechanism at the transcriptional level.

On the other hand, the results on the response to plant hormones confirmed that most of the genes examined were upregulated by ABA. However, except *CuAOγ1* encoding an apoplastic enzyme implicated in ABA-induced nitric oxide production [45] and *CuAOδ*, which encodes a putative vacuolar enzyme implicated in ABA-induced stomatal closure [21], genes for CuAOs showed no significant response to ABA. Although *CuAOζ* induction by ABA was previously reported [19], the only slight increase was detected under our experimental condition. Taken together with the result that all *PAO* genes except *PAO5* are upregulated by ABA, polyamine metabolism seems to be generally directed towards increasing intracellular accumulation of putrescine in the response to ABA. Many studies have revealed that putrescine levels are drastically increased by potassium deficiency [46], which also stimulates biosynthesis, accumulation, and transport of ABA [47,48].

The degree and time course of the response to NaSA and MeSA were different depending upon genes. Among other genes which generally responded more to NaSA than to MeSA, *ADC2* showed the highest fold induction at 6 h treatment with MeSA (Figure 1), suggesting different modes of action between NaSA and MeSA on *ADC2* expression. Increased expressions of *ADC1*, *ADC2*, *SAMDC1*, *SPMS*, and *PAO1* by 24 h treatment with NaSA are consistent with a recent study that showed a connection between SA signaling and polyamine metabolism [23]. The study also revealed that SA induces putrescine accumulation both in whole and apoplastic extracts. SA has also been shown to activate *ADC* and ornithine decarboxylase gene expression in in maize, tobacco, and tomato [49,50,51]. The putrescine accumulation might be explained in part by the reduction in *SAMDC2*, *CuAOα2*, *CuAOα3*, and *CuAOγ2* detected at 6 h treatment with NaSA. Expressions of *ARGH1* and *ARGH2* were not altered at 6 h treatment with NaSA but increased at 24 h, suggesting the involvement of additional factors in their regulation. We further found that treatment with ACC drastically induces *ADC2* expression but decreases *CuAOα2* expression. This may be in line with previous studies showing that ethylene enhances putrescine accumulation in some plants including rice [52] and that the *ADC2* promoter activity is reduced in an ethylene insensitive *etr1* mutant [6]. On the other hand, putrescine or polyamines are known to suppress ethylene production in many plant species. Contrasting regulations of *ADC2* and *CuAOα2* might be critical for the crosstalk between ethylene and polyamine metabolism. Overexpression of *ADC2* results in the accumulation of high levels of putrescine with no effect on spermidine and spermine contents and also affects gibberellin biosynthesis [53]. Moreover, *ADC2* expression was shown to be induced transiently by 2 h treatment with MeJA [4], although we detected no change after 6 h treatment (Table 2). Taken altogether, *ADC2* may serve as a hub connecting diverse functions of different plant hormones with putrescine.

## Figures and Tables

**Table 1:** Effect of polyamine treatment on polyamine metabolic genes.

Gene Name	Gene ID	Treatment (6 h)			
		Put 5 mM	Spd 1 mM	Spm 0.5 mM	Tspm 0.1 mM
*ADC1*	AT2G16500	1.25	0.50	1.17	1.30
*ADC2*	AT4G34710	5.90 *	0.14 *	1.07	0.60
*ARGAH1*	AT4G08900	1.46	1.04	1.36	0.74
*ARGAH2*	AT4G08870	1.51	1.10	1.04	1.18
*AIH*	AT5G08170	1.89	1.13	1.21	1.27
*CPA*	AT2G27450	1.33	1.20	1.21	0.89
*SPDS1*	AT1G23820	1.77	1.04	1.01	1.61
*SPDS2*	AT1G70310	1.17	1.85	1.20	0.91
*SPMS*	AT5G53120	1.49	1.90	1.49	0.85
*ACL5*	AT5G19530	1.10	2.33	1.34	0.28 **
*SAMDC1*	AT3G02470	1.14	0.96	1.17	0.94
*SAMDC2*	AT5G15950	1.63	1.42	1.30	0.87
*SAMDC3*	AT3G25570	1.06	1.43	1.26	0.74
*SAMDC4/BUD2*	AT5G18930	0.64	1.63	0.65	0.19 **
*PAO1*	AT5G13700	1.54	2.13	1.22	0.69
*PAO2*	AT2G43020	2.30	3.88 *	1.38	0.90
*PAO3*	AT3G59050	1.49	2.35	1.28	1.04
*PAO4*	AT1G65840	0.58	2.54	1.34	1.38
*PAO5*	AT4G29720	1.34	2.25	1.05	1.03
*CuAOα* *1*	AT1G31670	N.D.	N.D.	N.D.	N.D.
*CuAOα* *2*	AT1G31690	0.25	1.88	1.15	0.91
*CuAOα* *3/CuAO2*	AT1G31710	1.13	1.36	1.26	0.78
*CuAOβ* */ATAO1*	AT4G14940	0.48	1.39	1.45	0.61
*CuAOγ* *1/CuAO1*	AT1G62810	1.40	1.10	0.91	1.09
*CuAOγ* *2*	AT3G43670	0.69	0.95	1.36	0.87
*CuAOδ*	AT4G12270	0.90	1.34	2.40	0.35
*CuAOζ* */CuAO3*	AT2G42490	1.13	1.49	1.79	0.79
*UBQ10*	AT4G05320	0.98	0.95	0.94	0.92

Expression levels of three biological replicates each with duplicated PCR reactions are presented relative to the mock control. Significantly increased and decreased values evaluated by Student’s *t*-test are shaded with pale (* *p* < 0.05) or dark (** *p* < 0.01) green and red colors, respectively. N.D., not detected.

**Table 2:** Effect of plant hormone treatment on polyamine metabolic genes.

Gene Name	Gene ID	Treatment (6 h)					
		IAA 10 μM	Kinetin 5 μM	ABA 1 μM	ACC 10 μM	MeJA 2 μM	NaSA 1 mM
*ADC1*	AT2G16500	1.85	1.34	0.62	2.45	0.51	1.64
*ADC2*	AT4G34710	4.06 *	1.19	8.01 **	27.28 **	0.96	26.91 **
*ARGAH1*	AT4G08900	1.13	1.08	3.95 *	2.09	5.94 *	1.13
*ARGAH2*	AT4G08870	2.16	1.11	9.78 **	2.31	31.78 **	0.97
*AIH*	AT5G08170	1.66	0.59	5.24 *	1.34	1.65	3.23 *
*CPA*	AT2G27450	1.74	0.74	3.84 *	1.82	1.23	1.49
*SPDS1*	AT1G23820	1.14	1.72	1.55	1.36	1.07	1.40
*SPDS2*	AT1G70310	1.85	1.43	2.81 *	1.07	0.89	1.15
*SPMS*	AT5G53120	1.70	0.90	20.25 **	1.21	1.11	13.83 **
*ACL5*	AT5G19530	3.46 *	2.34	1.10	1.01	1.49	0.72
*SAMDC1*	AT3G02470	0.88	0.58	2.47 *	1.37	0.81	2.66
*SAMDC2*	AT5G15950	1.11	0.71	9.00 **	0.83	0.77	0.14
*SAMDC3*	AT3G25570	2.08	0.77	1.39	1.47	1.20	0.85
*SAMDC4/BUD2*	AT5G18930	2.95 *	0.92	2.15	1.41	0.91	0.40
*PAO1*	AT5G13700	3.51 *	1.09	5.28 *	2.73	0.73	8.00 **
*PAO2*	AT2G43020	0.72	1.05	3.16 *	1.71	1.16	1.25
*PAO3*	AT3G59050	2.38 *	0.83	2.39 *	2.10	2.46 *	1.11
*PAO4*	AT1G65840	1.04	0.56	3.25 *	1.14	0.66	0.86
*PAO5*	AT4G29720	2.87 *	1.52	2.03	1.73	1.88	0.33
*CuAOα* *1*	AT1G31670	N.D.	N.D.	N.D.	N.D.	N.D.	N.D.
*CuAOα* *2*	AT1G31690	0.19 *	1.58	1.45	0.03 **	1.17	0.11 **
*CuAOα* *3/CuAO2*	AT1G31710	0.32 *	1.61	0.89	0.88	3.84 *	0.30 *
*CuAOβ* */ATAO1*	AT4G14940	0.71	1.52	1.46	1.60	0.08	0.71
*CuAOγ* *1/CuAO1*	AT1G62810	1.38	0.70	10.10 **	1.57	0.79	0.74
*CuAOγ* *2*	AT3G43670	1.01	0.54	1.51	1.00	0.88	0.55 *
*CuAOδ*	AT4G12270	1.26	0.16	4.72 *	0.6	0.77	1.74
*CuAOζ* */CuAO3*	AT2G42490	0.84	0.76	1.91	1.91	1.22	2.45
*UBQ10*	AT4G05320	1.02	0.94	1.11	0.96	0.94	0.92

Expression levels of three biological replicates each with duplicated PCR reactions are presented relative to the mock control. Significantly increased and decreased values evaluated by Student’s *t*-test are shaded with pale (* *p* < 0.05) or dark (** *p* < 0.01) green and red colors, respectively. N.D., not detected.

## Supplementary Materials

The following are available online at https://www.mdpi.com/article/10.3390/cells10120000/s1, Table S1: Primer sequences used for qRT-PCR.
