# Supplementary material for: Responses of Polyamine-Metabolic Genes to Polyamines and Plant Stress Hormones in Arabidopsis Seedlings
Source: Cells. 2021 Nov 24;10(12):3283. doi: 10.3390/cells10123283 (PMC8699553; doi:10.3390/cells10123283)
Supplement: Supplementary file 1 [file cells-10-03283-s001.zip › cells-1416025-supplementary.pdf]

Table S1. Primer sequences used for qRT-PCR.

Gene name	Gene ID	Primer sequence	
		**Forward primer**	**Reverse primer**
ACT8	AT1G49240	5′-GTGAGCCAGATCTTCATTCGTC-3′	5′-TCTCTTGCTCGTAGTCGACAG-3′
UBQ10	AT4G05320	5′-CACACTCCACTTGGTCTTGC-3′	5′-AAGATCAACCTCTGCTGGTC-3′
ADC1	AT2G16500	5′-CCCATCGATACCTTTTCCAG-3′	5′-AAACCACTCGGATCTGTAAC-3′
ADC2	AT4G34710	5′-CCGCCGGTGATGTTTTTATC-3′	5′-CAATCCTAAACCACCGGAAG-3′
ARGAH1	AT4G08900	5′-CTTTTACAGGTTGGGATCAG-3′	5′-CCTGAAGGTTGTGTAAGATG-3′
ARGAH2	AT4G08870	5′-AAATGTTGGAAAACTTGAAACTAG-3′	5′-AAACTTAGCCGCGACCATTG-3′
AIH	AT5G08170	5′-TTCCTCATCACTCGGTTGTG-3′	5′-TTAATGAACGGCTCTCGTTC-3′
CPA	AT2G27450	5′-CTTCATTGCCGGACCAACAG-3′	5′-TAATGGATTTCCCCAGTGTG-3′
SPDS1	AT1G23820	5′-CATTTCTCGGAGATATTCACCAG-3′	5′-TCCATATGTTGCAGACTGGA-3′
SPDS2	AT1G70310	5′-GTCTCTCTTCTTCAATCCATG-3′	5′-TCCATATGTTGCAGACTGGA-3′
SPMS	AT5G53120	5′-ACATATCCAAGCGGCGTGAT-3′	5′-CCTCTTCAAGAGTTCTACAAAG-3′
ACL5	AT5G19530	5′-ACCGTTAACCAGCGATGCTT T-3′	5′-CCGTTAACTCTCTCTTTGATTC-3′
SAMDC1	AT3G02470	5′-GGTTTCTGGAAACAAGGAGT-3′	5′-TCTTGGAGCAGTTTGAGTAG-3′
SAMDC2	AT5G15950	5′-TCCTCTACATAAAAAGCGTG-3′	5′-GGAACCAGAGCTTTGAGAAA-3′
SAMDC3	AT3G25570	5′-CTGCTTACTCAAACATCACTG-3′	5′-GAACGAAGACTCTTTCCTTG-3′
SAMDC4/BUD2	AT5G18930	5′-CGAAGATATCTCTGAGGTGT-3′	5′-CTCCTTTTTTACTCCTCCGG-3′
PAO1	AT5G13700	5′-TGGTCAAGTAGCTGAAGAAG-3′	5′-CCCTAACTACCTGGTTTAGT-3′
PAO2	AT2G43020	5′-TAGGCCACAGGGTTACTAAG-3′	5′-ACTCTACTTTCGGCCAAAAG-3′
PAO3	AT3G59050	5′-GTTGGGATCAGGAAGAACTG-3′	5′-TGGAAGCTTTGGTTCGAATG-3′
PAO4	AT1G65840	5′-GAACCATAGGGTTACTAAAG-3′	5′-CCCGAGGAACTCCACATTAG-3′
PAO5	AT4G29720	5′-ACAACACCGACAAGGAGATT-3′	5′-CCAGTCTCTTTAGCGATTCT-3′
CuAOα1/CuAO5	AT1G31670	5′-GTTTGGTCCCAAAGGAATAG-3′	5′-GTTCCTGGCAGTGCACGTTG-3′
CuAOα2/CuAO8	AT1G31690	5′-GCTTGAGAGCGGAGGAGTTG-3′	5′-TTCTCTATTTCCCTATCTCTTTG-3′
CuAOα3/CuAO2	AT1G31710	5′-GTGTGGTCTCAAAGGAATAG-3′	5′-TTACAGTCCATCATCAAGTAG-3′
CuAOβ/ATAO1	AT4G14940	5′-GTATGGAGTAGTAGGAACAG-3′	5′-GTTTGACGTAACATAAGTCAATC-3′
CuAOγ1/CuAO1	AT1G62810	5′-GTCAGACCGGGACCGTTC-3′	5′-GGATATTTGATCTTCTCTACTTG-3′
CuAOγ2/CuAO7	AT3G43670	5′-AGTTTGGTCCGACAGAGATC-3′	5′-GCACAGTGTCGAATAAGATG-3′
CuAOδ/CuAO4	AT4G12270	5′-ATCACCGGTATACCGATAAG-3′	5′-AAAGTGACGTAGTGATCGTG-3′
CuAOζ/CuAO3	AT2G42490	5′-GTTTCACACTAATGCCACATG-3′	5′-AGTCTTGCAAACACACACAC-3′
